# The Association Between Mycobacteria-Specific Antigen-Induced Cytokines and Host Response to Latent Tuberculosis Infection Treatment in a Chinese Population

**DOI:** 10.3389/fmicb.2021.716900

**Published:** 2021-08-13

**Authors:** Xuefang Cao, Henan Xin, Haoran Zhang, Jianmin Liu, Shouguo Pan, Ying Du, Boxuan Feng, Zhusheng Quan, Ling Guan, Fei Shen, Zisen Liu, Dakuan Wang, Bin Zhang, Xueling Guan, Jiaoxia Yan, Qi Jin, Lei Gao

**Affiliations:** ^1^NHC Key Laboratory of Systems Biology of Pathogens, Center for Tuberculosis Research, Institute of Pathogen Biology, Peking Union Medical College, Chinese Academy of Medical Sciences, Beijing, China; ^2^The Sixth People’s Hospital of Zhengzhou, Zhengzhou, China; ^3^The Center for Disease Prevention and Control of Zhongmu County, Zhengzhou, China

**Keywords:** latent tuberculosis infection, preventive treatment, cytokines, IL-8, mycobacteria-specific

## Abstract

**Objectives:**

Exploring biomarkers monitoring latent tuberculosis infection (LTBI) treatment effectiveness would benefit optimizing the therapeutic regimen. This study aims to identify potential mycobacteria-specific antigen-induced cytokines associated with host responses to preventive treatment.

**Methods:**

Based on a randomized controlled trial on LTBI treatment among individuals with chest radiography abnormalities suggestive of prior tuberculosis (TB), the dynamically changed cytokine levels in QuantiFERON-TB Gold In-Tube (QFT) supernatants were estimated during the treatment by bead-based multiplex assays and enzyme-linked immunosorbent assay.

**Results:**

In total, 63 treated participants and 32 untreated controls were included in the study. The levels of 13 background-corrected mycobacteria-specific antigen-stimulated cytokines [basic fibroblast growth factor (FGF), growth-regulated oncogene (GRO)-α, interleukin (IL)-1α, IL-1ra, IL-12 (p70), stem cell factor (SCF), tumor necrosis factor-related apoptosis-inducing ligand (TRAIL), IL-8, interferon (IFN)-α2, IL-5, IL-12 (p40), leukemia inhibitory factor (LIF), and IL-17A] were found to be statistically different between before and after treatment in treated participants, while no statistically differences were observed in untreated controls. Among these 13 cytokines, the level of IL-8 was significantly lower in the QFT reversed group than that in the non-reversed group (*p* = 0.028) among treated participants, while such a difference was not found for untreated controls (*p* = 0.292).

**Conclusion:**

Our results suggested that the lower level of mycobacteria-specific antigen-induced IL-8 might be associated with the host’s positive response to LTBI treatment.

## Introduction

Approximately one-fourth of the world’s population were infected with *Mycobacterium tuberculosis* (*M.tb*), and there were more than 7 million new cases of active tuberculosis (TB) being reported in 2019 (World Health Organization, 2020a). It is estimated that 5–10% of individuals with latent tuberculosis infection (LTBI) might develop active disease during their lifetime. Promoting TB preventive treatment among high-risk populations with LTBI is one of the key tools to achieve the End TB Strategy targets as recommended by the World Health Organization (2020b). At present, assessing the decline of active TB incidence is a traditional way to evaluate the effect of preventive treatment that is usually influenced by multiple factors and that needs a long follow-up period and huge resource inputs. Therefore, identifying biomarkers promptly reflecting treatment effect is necessary, which might also provide insights into surrogate markers of protective immunity against TB.

Host immune cells secrete a number of cytokines and chemokine signals (Cooper, 2008; Chowdhury et al., 2014), which play active roles in the initiation and regulation of the immune response at various stages of disease development. Previous studies have reported several specific antigen-stimulated or unstimulated serum cytokine biomarkers for monitoring the potential effect of anti-TB treatment, such as tumor necrosis factor (TNF)-α, interleukin (IL)-10, IL-6, IL-1ra, macrophage inflammatory protein (MIP)-1β, IL-2/interferon (IFN)-γ, and IFN-inducible protein (IP)-10 (Cooper, 2008; Eum et al., 2010; Chowdhury et al., 2014). However, to date, few studies evaluated the usefulness of the cytokines in monitoring host response to LTBI treatment (Kabeer et al., 2011; Petruccioli et al., 2013). Although IFN-γ release assay (IGRA) is a valuable tool for establishing the diagnosis of LTBI, our recent study based on a randomized controlled trial (RCT) showed that similar decreased IFN-γ levels were observed in serial QuantiFERON-TB Gold In-Tube (QFT; a commercial IGRA) tests regardless if preventive treatment was initiated or not (Xin et al., 2020). Previous meta-analysis also validated our results that the IGRA was not suitable for monitoring host response to LTBI treatment (Clifford et al., 2015; Zhang et al., 2018). Therefore, our current study aimed to track mycobacteria-specific antigen-induced cytokine responses to LTBI treatment based on an RCT to identify potential biomarkers correlating with host response to preventive treatment.

## Materials and Methods

### Study Population

Study participants in the current study were selected from an RCT aiming to explore the effect of ultra-short regimen among rural residents with chest radiography abnormalities suggestive of prior TB lesions in China. Briefly, all participants with QFT (Qiagen, United States)-positive result (TB Ag-Nil ≥ 0.35 IU/ml) and without current active TB at baseline survey were included in the RCT. They were randomly classified into two groups [Intervention group: 6 weeks regimen of twice-weekly rifapentine (RPT) plus isoniazid (INH), with a maximum dose of 600 mg for each, between October 20, 2018, and November 30, 2018; Control group: without treatment). In the current study, 63 participants with available QFT supernatants who completed the assigned regimens and 32 subjects from the control group were randomly selected for cytokine measurements.

The protocol of the present study has been approved by the ethics committees of the Institute of Pathogen Biology, Chinese Academy of Medical Sciences (No.: IPB-2018-1). All participants have signed the written informed consent.

### Cytokine Measurements in the Supernatants of QuantiFERON-TB Gold In-Tube

Supernatants of QFT before the start of LTBI treatment (T0) and at 1 week post LTBI treatment (T1) have been collected and retained. Ready-made cytokine kit (Bio-Rad Laboratories, Hercules, CA, United States) that could simultaneously detect 48 cytokines [cutaneous T-cell attracting chemokine (CTACK), eotaxin, growth-regulated oncogene (GRO)-α, IP-10/C-X-C motif chemokine ligand (CXCL)10, macrophage chemotactic protein (MCP-1)/monocyte chemotactic and activating factor (MCAF), MCP-3, macrophage migration inhibitory factor (MIF), monokine induced by IFN-γ (MIG), MIP-1α, MIP-1β, platelet-derived growth factor (PDGF)-BB, Regulated upon Activation, Normal T Cell Expressed and Presumably Secreted (RANTES), stromal cell-derived factor (SDF)-1α, granulocyte-macrophage colony-stimulating factor (GM-CSF), G-CSF, stem cell factor (SCF), M-CSF, vascular endothelial growth factor (VEGF)-A, leukemia inhibitory factor (LIF), basic fibroblast growth factor (FGF), hepatocyte growth factor (HGF), β-nerve growth factor (NGF), stem cell growth factor (SCGF)-β, IFN-α2, IFN-γ, TNF-α, TNF-β, TNF-related apoptosis-inducing ligand (TRAIL), IL-1α, IL-1β, IL-1ra, IL-2Rα, IL-2, IL-3, IL-4, IL-5, IL-6, IL-7, IL-8, IL-9, IL-10, IL-12 (p70), IL-12 (p40), IL-13, IL-15, IL-16, IL-17A, and IL-18] were used. The levels of cytokines in the Nil supernatants (unstimulated) and the TBAg supernatants [stimulated with the mycobacteria-specific antigens: early secretory antigen target-6 (ESAT-6), culture filtrate protein 10 (CFP-10), and TB7.7] were analyzed by magnetic bead suspension array using the Bio-Plex Pro Human Cytokine panels according to the manufacturer’s instructions. Prior to measuring the samples, the supernatants were diluted 4× according to the manufacturers’ instructions. As the level of IL-8 was above the detection limit when measured for 4×-diluted supernatants by the Bio-Plex Pro Human Cytokine panels, the supernatants were diluted 100× and measured by an enzyme-linked immunosorbent assay (ELISA) (Human IL-8 Platinum ELISA kit #BMS2043; eBioscience, San Diego, CA). Cytokines with >50% of the samples below the lower detection level (LDL) or above the upper limit of the assay will be excluded from further statistical analysis. Additionally, cytokines with occasional values (<50%) below the LDL were assigned an averaged value between 0 and the lowest detectable level in each assay plate (Xin et al., 2019). Cytokines with occasional values (<50%) above the upper limit were assigned the upper limit (Ruhwald et al., 2008). Background-corrected mycobacteria-specific antigen-stimulated cytokine concentration was defined by subtracting the concentration in Nil supernatants from the corresponding concentration in TBAg supernatants.

### Statistical Analysis

Statistical analyses were performed using SAS 9.4 version (SAS Institute, Cary, NC) and GraphPad Prism 9 (GraphPad software, San Diego, CA). Participants who completed ≥90% doses of the therapy were defined as completed the regimens. In order to reflect the change of host infection status, a more strict definition of QFT reversion was used in the present study as IFN-γ level declined from >0.70 IU/ml at T0 to <0.20 IU/ml at T1 (van Zyl-Smit et al., 2009; Tagmouti et al., 2014; Nemes et al., 2017). Chi-square (χ^2^) test and Fisher’s exact test were used to compare the distribution of categorical variables across groups. The level of background-corrected mycobacteria-specific antigen-stimulated cytokine concentrations was presented with median (Q25–Q75). Wilcoxon rank sum test was used to compare responses between different participants at the same time point. Wilcoxon signed rank test was used to evaluate the responses for the same person at different time points. To assess the ability of the cytokines to reflect infection status, receiver operating characteristic (ROC) analysis was conducted, and the area under ROC curves (AUCs) was calculated. A two-tailed *p*-value < 0.05 was considered statistically significant.

## Results

### Characteristics of the Study Participants

A total of 32 untreated controls and 63 treated participants were included in this study. Table 1 shows major characteristics of the study participants. All of the study participants were HIV negative and without a history of diabetes. Roughly two-thirds of the treated and untreated participants were males, with a median age of 61 and 60 years, respectively. The majority of treated and untreated participants had a Bacille Calmette–Guérin (BCG) scar. No significant difference was found between the two groups with respect to age, gender, smoking, presence of TB history, and history of immunological diseases. In addition, there were no statistical differences in the IFN-γ levels between treated and untreated groups both at T0 (before the start of LTBI treatment) and T1 (at 1 week post LTBI treatment).

**TABLE 1:** Characteristics of the participants included in the study.

**Variables**	**Treated participants**	**Untreated controls**	***p*-value**
Total*	63	32	
**Age (median, Q25–Q75) (years)**	61.00 (50.00, 69.00)	60.00 (54.50, 68.00)	0.950^†^
**Gender, *n* (%)**			0.717^#^
Male	43 (68.25)	23 (71.88)	
Female	20 (31.75)	9 (28.12)	
**Smoking, *n* (%)**			0.606^#^
Never smoked	42 (66.67)	23 (71.88)	
Ever smoked	21 (33.33)	9 (28.12)	
**Presence of TB history, *n* (%)**			0.723^#^
No	54 (85.71)	29 (90.63)	
Yes	9 (14.29)	3 (9.38)	
**BCG scar, *n* (%)**			0.895^#^
Absent	10 (15.87)	4 (12.50)	
Present	53 (84.13)	28 (87.50)	
**Median BMI (Q25–Q75) (kg/m^2^)**	23.44 (21.51, 25.59)	24.24 (22.15, 25.70)	0.454^†^
**Median IFN-γ release of QFT (Q25–Q75) (IU/ml)**			
T0	2.16 (0.92, 4.67)	2.07 (0.66, 4.96)	0.909^†^
T1	0.66 (0.17, 3.10)	1.13 (0.15, 3.81)	0.491^†^
**Self-reported history of immunological diseases**			1.000^§^
No	61 (96.83)	31 (96.88)	
Yes	2 (3.17)	1 (3.12)	

*Q25, 25% quantile; Q75, 75% quantile; QFT, QuantiFERON-TB Gold In-Tube; T0, Before the start of LTBI treatment; T1, At 1 week post LTBI treatment; TB, tuberculosis; BCG, Bacille Calmette–Guérin; BMI, body mass index; IFN, interferon.*

*^∗^All study subjects were not infected with HIV and had no history of diabetes.*

*^†^*p* for Wilcoxon rank sum test.*

*^#^*p* for χ^2^ test.*

*^§^*p* for Fisher’s exact test.*

### Median Levels of Background-Corrected Mycobacteria-Specific Antigen-Stimulated Cytokines at Different Time Points

Table 2 shows the levels of the cytokines in different time points. As the levels of the two tested cytokines (MCP-1 and MIP-1α) by bead-based multiplex assays were below the LDL, they were not included for further data analysis.

**TABLE 2:** Median levels of background-corrected mycobacteria-specific antigen-stimulated cytokines at different time points among participants with and without preventive treatment.

**Cytokine**	**Cytokine levels in treated participants (*n* = 63) Median [Q25–Q75] (pg/ml)**			**Cytokine levels in untreated controls (*n* = 32) Median [Q25–Q75] (pg/ml)**		
	**T0**	**T1**	P* for difference	**T0**	**T1**	P* for difference
CTACK	−126.69 (−355.38, 310.37)	−207.32 (−416.83, 325.42)	0.113	−21.43 (−210.68, 281.68)	−190.66 (−444.45, −60.76)	<0.001
Eotaxin	−101.86 (−238.52, 0.60)	−107.95 (−242.26, 144.00)	0.782	−45.95 (−214.81, 423.54)	−334.25 (−517.47, −235.34)	<0.001
Basic FGF	81.04 (21.04, 292.26)	80.11 (−39.97, 158.40)	**<0.001**	−62.43 (−111.41, 10.46)	−31.45 (−59.91, −5.70)	0.202
G-CSF	519.58 (−1,659.44, 5,427.18)	−1,096.43 (−6,476.26, 4,077.80)	<0.001	−344.47 (−6,853.46, 4,148.10)	−6,565.22 (−7,795.16, −1,221.98)	0.009
GM-CSF	4.71 (−3.81, 22.90)	1.93 (−3.87, 15.40)	<0.001	−6.69 (−10.52, −0.32)	−2.53 (−3.58, 0.45)	0.006
GRO-α	809.51 (−1,265.36, 81,992.25)	595.50 (−1,134.21, 13,300.15)	**<0.001**	−988.19 (−5,966.80, 8,593.11)	−456.33 (−899.75, 0.00)	0.985
HGF	−148.07 (−582.83, 829.71)	−49.29 (−808.09, 658.56)	0.037	1.88 (−844.65, 371.83)	−532.48 (−788.68, −338.72)	0.021
IFN-α2	26.16 (5.19, 124.89)	27.52 (−3.38, 79.35)	**<0.001**	−1.30 (−22.00,9.35)	−5.88 (−17.48, 7.55)	0.661
IFN-γ	121.89 (−182.46, 1,170.19)	−10.67 (−283.85, 776.98)	<0.001	85.98 (−14.39, 613.95)	55.75 (−118.75, 196.54)	0.018
IL-1α	120.76 (−5.66, 421.61)	88.43 (−12.61, 323.20)	**0.006**	−45.27 (−133.76, 61.05)	−13.23 (−67.36, 34.49)	0.253
IL-1β	100.06 (−37.61, 561.80)	131.44 (−91.44, 395.38)	0.448	81.01 (−92.19, 289.87)	76.33 (−39.97, 346.43)	0.358
IL-1ra	5,313.52 (2,238.55, 10,237.62)	3,281.28 (1,399.17, 6,058.60)	**0.002**	1,636.99 (−1,535.85, 3,162.22)	−480.65 (−1,726.70, 856.02)	0.066
IL-2	162.99 (34.15, 886.96)	52.65 (4.48, 267.25)	<0.001	109.41 (27.75, 255.30)	23.40 (−4.20, 52.59)	<0.001
IL-2Rα	41.57 (−22.20, 294.21)	66.28 (−133.81, 270.83)	0.043	−34.95 (−189.65, 84.15)	−95.49 (−169.82, −63.47)	0.016
IL-3	2.86 (0.71, 6.51)	0.76 (0.07, 2.94)	0.017	1.08 (−0.11, 2.06)	4.55 (0.07, 8.91)	0.002
IL-4	3.84 (−0.78, 16.87)	3.02 (−1.59, 9.17)	<0.001	0.80 (−3.64, 3.43)	−2.11 (−3.99, −0.47)	0.005
IL-5	63.47 (27.45, 220.79)	65.48 (22.19, 153.36)	**<0.001**	−5.82 (−31.96, 29.86)	−12.51 (−39.10, 0.00)	0.348
IL-6	418.17 (−475.29, 1,923.64)	409.00 (−7.01, 984.98)	0.675	−86.44 (−1,203.28, 772.15)	31.75 (−69.02, 182.86)	0.409
IL-7	1.34 (−1.63, 4.26)	2.30 (−9.46, 5.38)	0.105	0.95 (−5.60, 14.35)	−8.98 (−11.18, −5.64)	<0.001
IL-9	9.32 (−64.26, 254.14)	−12.47 (−121.843, 144.24)	<0.001	0.18 (−98.14, 114.11)	−128.29 (−171.28, −69.85)	<0.001
IL-10	0.11 (−4.84, 10.26)	2.91 (−5.32, 8.92)	0.992	1.69 (−8.06, 17.48)	−6.62 (−11.93, −4.31)	<0.001
IL-12 (p70)	1.42 (−2.78, 17.64)	0.00 (−11.98, 9.97)	**<0.001**	−2.85 (−9.75, 1.91)	1.04 (−1.35, 2.66)	0.170
IL-12 (p40)	123.97 (−13.53, 715.25)	140.63 (−164.59, 481.72)	**<0.001**	−173.10 (−235.07, 54.61)	−83.20 (−144.59, −19.47)	0.784
IL-13	4.97 (−0.59, 15.45)	2.01 (−0.58, 7.27)	<0.001	4.195 (0.00, 9.28)	−1.76 (−3.07, −0.59)	<0.001
IL-15	0.29 (−164.50, 715.55)	1.48 (−286.59, 485.34)	<0.001	−233.59 (−395.40, −145.09)	−115.22 (−115.22, −32.60)	0.036
IL-16	−138.75 (−291.14, 350.07)	−72.56 (−560.79, 350.35)	0.019	−276.05 (−471.36, −120.71)	143.17 (−209.84, −88.70)	<0.001
IL-17A	25.43 (4.46, 73.11)	27.16 (−7.34, 51.39)	**0.009**	−12.11 (−38.63, 10.30)	−19.11 (−26.51, −7.88)	0.475
IL-18	1.88 (−16.01, 59.88)	14.89 (−23.28, 43.01)	0.075	−5.81 (−24.21, 25.15)	−11.32 (−24.39, −4.94)	0.029
IP-10	7,671.40 (2,985.82, 31,427.30)	3,728.97 (380.94, 12,104.73)	<0.001	25,865.84 (12,629.40, 51,161.97)	14,242.36 (5,127.39, 28,061.75)	<0.001
LIF	38.99 (−49.93, 414.39)	53.35 (−53.31, 211.77)	**0.039**	−102.02 (−164.07, 23.89)	−47.10 (−79.15, −17.43)	0.159
MCP-3	237.46 (−491.24, 1,413.60)	106.61 (−122.82, 614.29)	0.030	891.22 (−33.25, 2,340.39)	66.49 (−136.80, 393.31)	0.007
M-CSF	14.46 (−3.25, 48.34)	23.95 (−14.06, 40.14)	0.095	−5.98 (−27.06, 17.98)	−17.72 (−29.72, −12.12)	0.007
MIF	−4,463.78 (−18,742.85, 54,602.15)	−4,716.22 (−32,368.19, 32,609.38)	0.012	−6,569.46 (−39,253.70, 79,184.35)	79,184.35 (−278,218.00, −61,434.70)	<0.001
MIG	10,868.28 (2,936.25, 43,695.62)	4,087.57 (−1,437.25, 15,899.27)	<0.001	24,793.82 (8,204.65, 35,168.92)	6,507.82 (849.08, 16,911.68)	<0.001
MIP-1β	808.92 (−638.27, 2,166.50)	632.93 (−113.95, 1,511.35)	0.444	184.00 (−1,383.81, 2,326.30)	−493.34 (−1,835.02, −49.73)	0.020
β-NGF	0.99 (−4.52, 14.13)	1.44 (−2.54, 7.35)	0.124	−1.34 (−7.11, 1.02)	−3.78 (−5.60, −1.60)	0.302
PDGF-BB	33.27 (−1,082.29, 2,226.18)	−163.05 (−1,278.39, 984.52)	0.126	548.28 (−341.00, 5,473.46)	−712.68 (−3,272.68, 447.00)	<0.001
RANTES	1,082.81 (−4,462.73, 18,580.12)	4,817.44 (−2,040.70, 12,509.52)	0.626	−3,178.03 (−6,311.22, 5,383.64)	−9,457.21 (−15,871.87, −3,710.35)	0.002
SCF	42.33 (−75.85, 310.61)	20.61 (−55.24, 168.30)	**0.010**	−36.71 (−143.19, 57.44)	−32.29 (−67.71, −1.35)	0.855
SCGF-β	16,978.97 (4,901.23, 31,760.59)	22,843.18 (8,420.48, 41,655.35)	0.444	13,478.77 (−1,194.48, 55,445.01)	−11,191.30 (−40,922.10, 3,813.70)	<0.001
SDF-1α	−232.05 (−376.06, 951.00)	−147.56 (−1,065.19, 867.26)	<0.001	−48.70 (−469.39, 1,070.00)	−519.76 (−751.96, −224.45)	0.001
TNF-α	731.72 (301.32, 1,621.36)	746.67 (281.86, 1,165.15)	0.428	−251.12 (−452.22, 257.03)	−8.81 (−258.99, 190.47)	0.648
TNF-β	218.02 (118.55, 295.88)	231.66 (175.97, 286.04)	0.374	6.90 (−0.18, 363.38)	−168.20 (−221.98, −101.96)	<0.001
TRAIL	214.15 (−147.40, 756.54)	136.19 (−26.94, 374.05)	**<0.001**	73.92 (−239.17, 239.09)	22.19 (−56.78, 97.07)	0.913
VEGF-A	−261.63 (−373.92, 468.14)	−247.68 (−554.52, 341.60)	<0.001	−252.99 (−398.50, −107.53)	−111.33 (−169.53, −52.10)	0.003
IL-8	12,242.90 (1,103.07, 37,141.72)	4,604.91 (−618.26, 16,283.70)	**0.002**	9,470.20 (−627.99, 24,930.28)	6,978.31 (3,812.93, 17,469.12)	0.784

QFT, QuantiFERON-TB Gold In-Tube; T0, Before the start of LTBI treatment; T1, At 1 week post LTBI treatment; Q25, 25% quantile; Q75, 75% quantile.

*Wilcoxon signed rank test; CTACK, cutaneous T cell-attracting chemokine; GRO, growth-regulated oncogene; TNF, tumor necrosis factor; MCP, macrophage chemotactic protein; MIF, macrophage migration inhibitory factor; MIG, monokine induced by IFN-γ; PDGF, plateletderived growth factor; RANTES, Regulated upon Activation, Normal T Cell Expressed and Presumably Secreted; SDF, stromal cell-derived factor; GM-CSF, granulocyte-macrophage colony-stimulating factor; SCF, stem cell factor; VEGF, vascular endothelial growth factor; LIF, leukemia inhibitory factor; FGF, fibroblast growth factor; HGF, hepatocyte growth factor; NGF, nerve growth factor; SCGF, stem cell growth factor; TRAIL, TNF-related apoptosis-inducing ligand; IL, interleukin; MIP, macrophage inflammatory protein; IFN, interferon; IP, IFN-inducible protein.

*Bold values correspond to cytokines related to preventive treatment, which were identified like this: compared to T0, changes were statistically significant for cytokines at T1 in treated participants, while there was no difference in levels of cytokines between T0 and T1 in untreated controls.*

**FIGURE 1:**
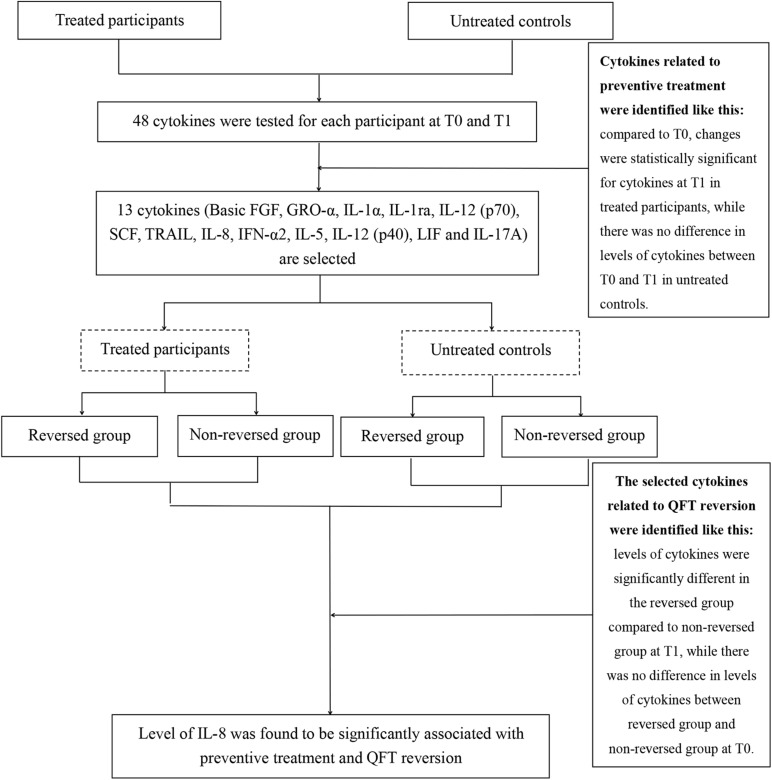
Flowchart to identify the relation between cytokine levels and preventive treatment efficacy. A total of 63 treated participants and 32 untreated controls were included in the present study. Here, 13 of 48 cytokines changed significantly from T0 [before the start of latent tuberculosis infection (LTBI) treatment] to T1 (at 1 week post LTBI treatment) in treated participants but not in untreated controls. Further analysis of the relationship between the selected cytokines and infection status. Only the level of interleukin (IL)-8 was significantly different in the reversed group compared with the non-reversed group at T1 in treated participants.

The definition used to identify background-corrected mycobacteria-specific antigen-stimulated cytokines related to preventive treatment was shown in Figure 1. According to this definition, compared with the level at T0, there were statistically significant decreases in levels of basic FGF, GRO-α, IL-1α, IL-1ra, IL-12 (p70), SCF, TRAIL, and IL-8 and increases in IFN-α2, IL-5, IL-12 (p40), LIF, and IL-17A at T1 in treated participants (Table 2). However, in untreated participants, as compared with the level at T0, there was no statistically significant difference being observed in the levels of the above cytokines at T1 (Table 2).

### Median Levels of the Selected Background-Corrected Mycobacteria-Specific Antigen-Stimulated Cytokines Classified by QuantiFERON-TB Gold In-Tube Results

**FIGURE 2:**
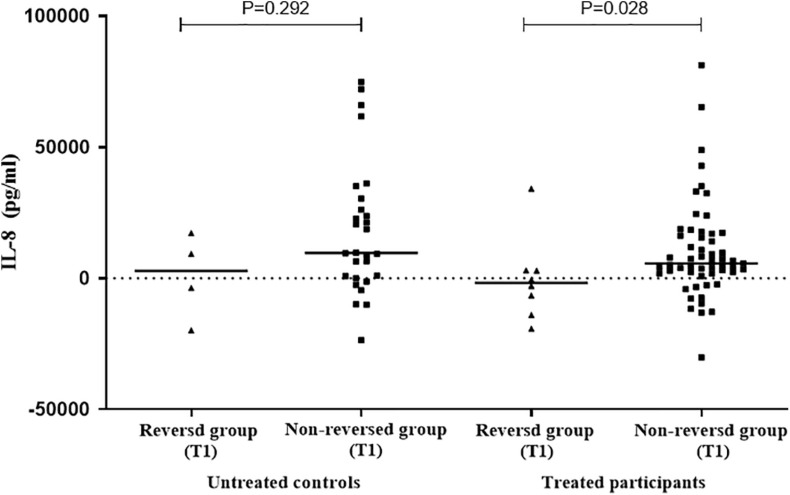
Distribution of the levels of background-corrected mycobacteria-specific antigen-stimulated IL-8 among treated and untreated participants at T1. According to QuantiFERON-TB Gold In-Tube (QFT) result at T1 [at 1 week post LTBI treatment], the treated and untreated participants were both divided into two groups (reversed group and non-reversed group). For treated participants, the level of IL-8 was significantly lower in the reversed group compared to that in the non-reversed group at T1. For untreated participants, there was no significant difference in the level of IL-8 between the two groups at T1.

In order to further explore the relationship between the selected cytokines and host infection status (the level of released IFN-γ in the QFT test was used to reflect infection status), the treated and untreated participants were both divided into two groups according to their QFT retesting results at T1 (reversed or not reversed) (Figure 1). As shown in Figure 2 and Table 3, for treated participants, only the level of background-corrected mycobacteria-specific antigen-stimulated IL-8 was significantly lower in the reversed group as compared to that in the non-reversed group at T1. However, there was no significant difference in the level of the cytokine between the two groups at T0. For untreated participants, there was no significant difference in the level of IL-8 between the two groups at both T0 and T1 (Table 4).

**TABLE 3:** Median levels of the selected background-corrected mycobacteria-specific antigen-stimulated cytokines classified by the value of QFT results among treated participants.

**Cytokine**	**Treated participants (*n* = 63)**					
	**Cytokine levels at T0 Median [Q25–Q75] (pg/ml)**			**Cytokine levels at T1 Median [Q25–Q75] (pg/ml)**		
	**Non-reversed (*n* = 55)**	**Reversed (*n* = 8)**	***P*# for difference**	**Non-reversed (*n* = 55)**	**Reversed (*n* = 8)**	***P*^#^ for difference**
Basic FGF	81.04 (12.94, 276.97)	145.22 (67.88, 328.15)	0.348	68.08 (−73.96, 158.40)	95.19 (71.50, 160.15)	0.244
GRO-α	519.97 (−1,472.48, 71,627.55)	42,047.20 (−716.50, 230,889.60)	0.529	459.98 (−1,491.71, 16,020.80)	3,421.71 (1,482.65, 13,042.02)	0.135
IFN-α2	23.01 (2.34, 124.27)	63.27 (21.60, 132.83)	0.288	19.71 (−11.17, 67.53)	39.16 (25.94, 84.27)	0.197
IL-1α	97.05 (−10.07, 421.61)	301.88 (26.35, 465.80)	0.476	80.18 (−38.18, 290.17)	254.26 (94.20, 350.17)	0.124
IL-1ra	5,143.59 (1,298.25, 10,237.62)	6,817.13 (4,253.17, 11,206.12)	0.415	3,852.98 (1,376.96, 7,467.87)	3,018.15 (2,316.23, 3,800.02)	0.672
IL-5	60.76 (23.53, 206.45)	130.78 (60.93,252.10)	0.190	53.69 (14.20, 153.36)	78.03 (58.10, 139.78)	0.279
IL-12 (p70)	1.84 (−2.32, 13.22)	−0.03 (−3.07, 20.58)	0.726	−0.20 (−13.33, 9.97)	2.08 (0.27, 7.833)	0.193
IL-12 (p40)	123.97 (−31.39, 622.58)	312.16 (27.64, 829.71)	0.642	119.93 (−238.93, 489.65)	194.30 (126.21, 473.19)	0.337
IL-17A	25.43 (3.25, 73.11)	31.64 (17.94, 90.72)	0.489	21.46 (−13.95, 55.76)	32.81 (25.65, 41.56)	0.337
LIF	38.99 (−57.79, 394.28)	130.55 (−8.81, 449.10)	0.439	53.35 (−64.83, 211.776)	47.20 (19.78, 228.92)	0.543
SCF	25.31 (−75.85, 310.61)	140.15 (−48.96, 307.95)	0.703	20.00 (−89.42, 185.11)	60.73 (−21.95, 118.97)	0.543
TRAIL	204.34 (−155.68, 756.54)	351.16 (193.24, 797.38)	0.415	191.025 (−121.07, 487.18)	197.86 (28.72, 311.18)	1.000
IL-8	14,104.95 (1,052.14, 35,418.65)	9,755.15 (2,196.48, 39,288.45)	0.975	5,621.42 (1,831.40, 17,000.41)	−1,756.25 (−10,297.41, 2,954.17)	**0.028**

*QFT, QuantiFERON-TB Gold In-Tube; T0, Before the start of LTBI treatment; T1, At 1 week post LTBI treatment; Q25, 25% quantile; Q75, 75% quantile; GRO, growth-regulated oncogene; SCF, stem cell factor; LIF, leukemia inhibitory factor; FGF, fibroblast growth factor; TRAIL, TNF-related apoptosis-inducing ligand; IL, interleukin; IFN, interferon.*

*^#^Wilcoxon rank sum test.*

**TABLE 4:** Median levels of the selected background-corrected mycobacteria-specific antigen-stimulated cytokines classified by the value of QFT results at T1 among untreated participants.

**Cytokine**	**Untreated participants (*n* = 32)**					
	**Cytokine levels at T0 Median [Q25–Q75] (pg/ml)**			**Cytokine levels at T1 Median [Q25–Q75] (pg/ml)**		
	**Non-reversed (*n* = 28)**	**Reversed (*n* = 4)**	***P*** ^#^ **for difference**	**Non-reversed (*n* = 28)**	**Reversed (*n* = 4)**	***P*** ^#^ **for difference**
Basic FGF	−50.31 (−95.83, 11.85)	−121.17 (−142.33, −70.82)	0.093	−31.75 (−59.91, −11.53)	−16.53 (−52.63, 10.75)	0.588
GRO-α	−525.93 (−2,499.48, 21,403.38)	−22,322.45 (−31,132.96, −12,086.71)	0.008	−456.33 (−899.75, −72.89)	−399.93 (−1,120.60, 289.82)	0.798
IFN-α2	−0.44 (−19.40, 15.28)	−24.88 (−28.98, −18.25)	0.049	−5.88 (−17.48, 7.55)	−2.35 (−23.75, 14.27)	0.977
IL-1α	3.88 (−129.60, 74.28)	−260.31 (−454.41, −28.72)	0.221	−13.23 (−67.36, 24.70)	−4.94 (−65.77, 42.74)	0.932
IL-1ra	1,852.02 (126.15, 3,720.75)	−3,519.67 (−6,811.04, −1,546.90)	0.005	−335.88 (−1,726.70, 856.02)	−750.39 (−2,058.43, 232.53)	0.550
IL-5	6.35 (−30.33, 33.15)	−75.75 (−81.14, −49.11)	0.018	−20.42 (−39.10, −2.07)	−3.25 (−35.73, 5.46)	0.732
IL-12 (p70)	−2.85 (−9.54, 1.91)	−5.86 (−13.36, 13.29)	0.776	1.06 (−0.83, 3.15)	−0.27 (−2.64, 1.57)	0.305
IL-12 (p40)	−130.44 (−209.32, 59.41)	−241.56 (−336.59, −204.68)	0.064	−83.20 (−144.59, −26.97)	−61.35 (−191.67, 15.72)	0.754
IL-17A	−8.37 (−33.59, 12.08)	−44.28 (−48.33, −42.05)	0.015	−19.11 (−26.51, −9.81)	−12.66 (−26.75, 2.15)	0.476
LIF	−87.72 (−137.34, 27.11)	−203.63 (−256.63, −157.02)	0.018	−47.10 (−79.15, −18.23)	−39.55 (−89.48, −0.90)	0.887
SCF	−15.17 (−79.04, 60.41)	−167.55 (−179.76, −149.18)	0.021	−42.33 (−67.71, 6.05)	−22.04 (−68.63, −12.66)	0.932
TRAIL	109.39 (−207.65, 283.72)	−232.74 (−605.18, 47.33)	0.082	22.19 (−51.64, 112.54)	21.12 (−169.83, 87.60)	0.711
IL-8	9,682.43 (394.28, 28,275.35)	2,830.40 (−11,746.80, 13,275.50)	0.181	6,978.30 (4,410.19, 21,850.52)	6,352.37 (−3,130.43, 10,772.31)	0.292

*QFT, QuantiFERON-TB Gold In-Tube; T0, Before the start of LTBI treatment; T1, At 1 week post LTBI treatment; Q25, 25% quantile; Q75, 75% quantile; GRO, growth-regulated oncogene; SCF, stem cell factor; LIF, leukemia inhibitory factor; FGF, fibroblast growth factor; TRAIL, TNF-related apoptosis-inducing ligand; IL, interleukin; IFN, interferon.*

*^#^Wilcoxon rank sum test.*

### Performance of Background-Corrected Mycobacteria-Specific Antigen-Stimulated IL-8 on Reflecting Infection Status

**FIGURE 3:**
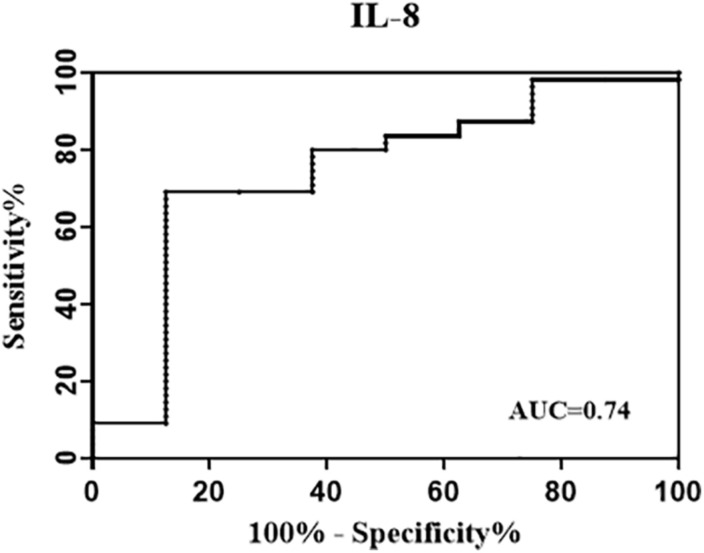
Receiver operating characteristic (ROC) curve evaluating the performance of background-corrected mycobacteria-specific antigen-stimulated IL-8 in reflecting infection status. The treated participants were divided into two groups according to their QFT status at T1 [at 1 week post LTBI treatment] (reversed group and non-reversed group). The ROC analysis was performed to evaluate the performance of IL-8 in identifying QFT reversion, and the area under the ROC curves (AUC) for IL-8 was 0.74.

To further evaluate the performance of background-corrected mycobacteria-specific antigen-stimulated IL-8 in reflecting infection status reflected by a more strict definition of reversion, the ROC analysis was performed and the AUC was calculated among treated participants at T1. As shown in Figure 3, the AUC for IL-8 was 0.74.

## Discussion

Based on a randomized controlled study, the level of 13 cytokines [basic FGF, GRO-α, IL-1α, IL-1ra, IL-12 (p70), SCF, TRAIL, IL-8, IFN-α2, IL-5, IL-12 (p40), LIF, and IL-17A] induced by mycobacteria-specific antigen was changed significantly after LTBI treatment. Notably, IL-8 might be a potential marker to reflect the change of TB infection status, indicating that it might play an important role in host immune protection against TB infection.

There have been several studies illustrating the importance of cytokines as a biomarker for monitoring the anti-TB or LTBI preventive treatment. A prospective study by Clifford et al. (2017) observed a statistically significant decline in IL-1ra (CFP-10, ESAT-6, PPD), IL-2 (CFP-10, ESAT-6), and IP-10 (CFP-10, ESAT-6) responses after treatment with INH in participants with LTBI. Our previous study in which samples from LTBI participants were unstimulated with mycobacteria-specific antigen also reported a decrease in IL-1ra responses after treatment and additionally reported that serum IL-1ra was statistically higher among those who developed active disease during follow-up compared with those who stayed healthy (Zhang et al., 2020). A similar finding was observed in our current study, which found the level of IL-1ra decline after preventive treatment for LTBI, suggesting the possibility that it might be used for real-time treatment effect monitoring. In addition, a prospective study conducted in active TB cases reported that all antigens induced higher levels of TNF-α and IL-17 and low levels of IL-10 and sIL-2R-α before treatment and after 2 weeks of treatment (Mensah et al., 2014). These studies consistently suggested that mycobacteria-specific cytokines might be potential correlates of successful treatment of both active TB and LTBI.

Assessing the effect of preventive treatment promptly by biomarkers remains challenging yet is crucial to TB control. For active TB, the current standard for use as a surrogate treatment effect remains focused on culture conversion supplemented by clinical and radiological improvement. However, for LTBI without symptoms or signs, exploring the change of blood biomarkers indicating changing of *M.tb* infection status is one way to reflect the response to preventive treatment, facilitate treatment monitoring, and individualize treatment regimen adjustment. A review has suggested a clearance model of *M.tb* based on animal and *ex vivo* human experiments (Verrall et al., 2014) that showed that, in addition to immune clearance by early and delayed clearance, *M.tb* could be eliminated from host tissue by anti-TB drugs. A subsequent study used an *in vitro* model of treated *M.tb* infection coupled with gel-enhanced liquid chromatography–tandem mass spectrometry (GeLC MS/MS) to identify and quantify peptides that may indicate *M.tb* infection clearance and found that several activated proteins were associated with several mechanisms against *M.tb* infection and could potentially be used as biomarkers for TB treatment monitoring (Kaewseekhao et al., 2015).

In this study, we used a more rigorous definition for QFT reversion to reflect infection status and found that the level of IL-8 was lower in the reversed group than that in the non-reversed group after treatment in the treated group. IL-8 was firstly isolated from monocytes as a neutrophil attractant (Schröder et al., 1987; Walz et al., 1987; Yoshimura et al., 1987). It is a CXC chemokine that is also chemotactic for T lymphocytes (Larsen et al., 1989; Gerszten et al., 1999). Monocytes and macrophages infected with *M.tb* may be primary producers of IL-8 during the course of developing active disease (Zhang et al., 1995; Riedel and Kaufmann, 1997; Ameixa and Friedland, 2002), and neutrophils and respiratory epithelial cells also have the capability to secrete this chemokine (Lin et al., 1998; Wickremasinghe et al., 1999; Silva Miranda et al., 2012). Previous studies have shown that epithelial cells secrete IL-8 and other chemokines following infection by pathogenic respiratory viruses (Fiedler et al., 1995; Subauste et al., 1995) and bacteria (DiMango et al., 1995; Naumann et al., 1997). IL-8 mainly exhibits two main biological activities: chemical attraction and activation of several types of white blood cells. These characteristics can have important clinical consequences by affecting the pathogenesis of infectious diseases, such as TB and coronavirus disease 2019 (COVID-19) (Krupa et al., 2015; Del Valle et al., 2020). Besides, IL-8 was ever proven to play a central role in normal immune response to *M.tb* and had been shown to be absolutely required for granuloma formation (Silva Miranda et al., 2012). It also had the ability to directly interact with *M.tb* and in this way enhance antimicrobial functions of pro-inflammatory cells, that is, macrophages and neutrophils (Krupa et al., 2015). Moreover, our previous study found that the serum level of IL-8 was dramatically lower in the QFT reversion group than that in the QFT persistent positive group (Xin et al., 2019). Based on the above evidence, decreased IL-8 level in our study might reflect reduced bacterial replication activity caused by preventive treatment and further suggested a predisposing role of IL-8 as a biomarker to indicate *M.tb* infection clearance. Furthermore, the level of mycobacteria-specific antigen-induced IL-8 was anticipated to be more sensitive to the change of *M.tb* infection status compared to the level in peripheral blood.

When interpreting the results of the study, several limitations should be kept in mind. First, although we used the same Luminex kit to test QFT supernatants collected at different time points, the influence of inconsistent sample quality on testing results could not be completely ruled out. Second, as the target population of the RCT was those with chest radiography abnormalities suggestive of prior TB lesions, generalization of the study results should be with caution. Third, previous studies reported that decreased IFN-γ levels in the QFT test should not be used for monitoring host response to LTBI treatment. Therefore, we conducted this study to identify alternative biomarkers reflecting preventive treatment effect. Although we used a more strict definition of QFT reversion (from IFN-γ > 0.7 IU/ml at T0 to <0.2 IU/ml at T1) to reflect infection clearance, misclassification still could not be excluded.

## Conclusion

Our results suggested that the level of mycobacteria-specific antigen-induced IL-8 decreased along with preventive treatment and might be used to identify infection clearance suggested by a strict definition of QFT reversion. It provides a clue for exploring prognosis biomarkers to evaluate the performance of LTBI treatment.

## Data Availability Statement

The raw data supporting the conclusions of this article will be made available by the authors, without undue reservation.

## Ethics Statement

The studies involving human participants were reviewed and approved by the protocol of the present study has been approved by the Ethics Committees of the Institute of Pathogen Biology, Chinese Academy of Medical Sciences (No: IPB-2018-1). All participants have signed the written informed consent. The patients/participants provided their written informed consent to participate in this study.

## Author Contributions

LeG and QJ designed the study. XC, HX, HZ, SP, ZL, DW, JY, BF, ZQ, YD, BZ, JL, LiG, FS, and XG were in charge of data management. XC, HX, and LeG did data analysis and wrote the report. QJ commented on the report and improved English writing. SP, HX, ZL, DW, JY, BF, ZQ, YD, JL, BZ, LiG, FS, and XG organized investigations at the study sites. All authors contributed to the review and revision and have seen and approved the final version of the manuscript.

## Conflict of Interest

The authors declare that the research was conducted in the absence of any commercial or financial relationships that could be construed as a potential conflict of interest.

## Publisher’s Note

All claims expressed in this article are solely those of the authors and do not necessarily represent those of their affiliated organizations, or those of the publisher, the editors and the reviewers. Any product that may be evaluated in this article, or claim that may be made by its manufacturer, is not guaranteed or endorsed by the publisher.

## References

[B1] Ameixa C. Friedland J. S. (2002). Interleukin-8 secretion from *Mycobacterium* tuberculosis-infected monocytes is regulated by protein tyrosine kinases but not by ERK1/2 or p38 mitogen-activated protein kinases. *Infect. Immun.* 70 4743–4746. 10.1128/IAI.70.8.4743-4746.2002 12117995 PMC128139

[B2] Chowdhury I. H. Ahmed A. M. Choudhuri S. Sen A. Hazra A. Pal N. K. (2014). Alteration of serum inflammatory cytokines in active pulmonary tuberculosis following anti-tuberculosis drug therapy. *Mol. Immunol.* 62 159–68. 10.1016/j.molimm.2014.06.002 25019566

[B3] Clifford V. He Y. Zufferey C. Connell T. Curtis N. (2015). Interferon gamma release assays for monitoring the response to treatment for tuberculosis: a systematic review. *Tuberculosis (Edinb)* 95:639e50. 10.1016/j.tube.2015.07.002 26515270

[B4] Clifford V. Tebruegge M. Zufferey C. Germano S. Forbes B. Cosentino L. (2017). Mycobacteria-specific cytokine responses as correlates of treatment response in active and latent tuberculosis. *J. Infect.* 75 132–145. 10.1016/j.jinf.2017.04.011 28483404

[B5] Cooper A. M. (2008). The role of cytokines in the initiation, expansion, and control of cellular immunity to tuberculosis. *Immunol. Rev.* 226 191–204. 10.1111/j.1600-065X.2008.00702.x 19161425 PMC4298252

[B6] Del Valle D. M. Kim-Schulze S. Huang H. H. Beckmann N. D. Nirenberg S. Wang B. (2020). An inflammatory cytokine signature predicts COVID-19 severity and survival. *Nat. Med.* 26 1636–1643. 10.1038/s41591-020-1051-9 32839624 PMC7869028

[B7] DiMango E. Zar H. J. Bryan R. Prince A. (1995). Diverse *Pseudomonas aeruginosa* gene products stimulate respiratory epithelial cells to produce interleukin-8. *J. Clin. Invest.* 96 2204–2210. 10.1172/JCI118275 7593606 PMC185870

[B8] Eum S. Y. Lee Y. J. Min J. H. Kwak H. K. Hong M. S. Kong J. H. (2010). Association of antigen-stimulated release of tumor necrosis factor-alpha in whole blood with response to chemotherapy in patients with pulmonary multidrug-resistant tuberculosis. *Respiration* 80 275–284. 10.1159/000283687 20145387 PMC2955738

[B9] Fiedler M. A. Wernke-Dollries K. Stark J. M. (1995). Respiratory syncytial virus increases IL-8 gene expression and protein release in A549 cells. *Am. J. Physiol.* 269 L865–L872. 10.1152/ajplung.1995.269.6.L865 8572249

[B10] Gerszten R. E. Garcia-Zepeda E. A. Lim Y. C. Yoshida M. Ding H. A. Gimbrone M. A. Jr. (1999). MCP-1 and IL-8 trigger fifirm adhesion of monocytes to vascular endothelium under flflow conditions. *Nature* 398 718–723. 10.1038/19546 10227295

[B11] Kabeer B. S. Raja A. Raman B. Thangaraj S. Leportier M. Ippolito G. (2011). IP-10 response to RD1 antigens might be a useful biomarker for monitoring tuberculosis therapy. *BMC Infect. Dis.* 11:135. 10.1186/1471-2334-11-135 21595874 PMC3120672

[B12] Kaewseekhao B. Naranbhai V. Roytrakul S. Namwat W. Paemanee A. Lulitanond V. (2015). Comparative proteomics of activated THP-1 cells infected with *Mycobacterium tuberculosis* identifies putative clearance biomarkers for tuberculosis treatment. *PLoS One* 10:e0134168. 10.1371/journal.pone.0134168 26214306 PMC4516286

[B13] Krupa A. Fol M. Dziadek B. R. Kepka E. Wojciechowska D. Brzostek A. (2015). Binding of CXCL8/IL-8 to *Mycobacterium tuberculosis* modulates the innate immune response. *Mediators Inflamm.* 2015:124762. 10.1155/2015/124762 26300588 PMC4537748

[B14] Larsen C. G. Anderson A. O. Appella E. Oppenheim J. J. Matsushima K. (1989). The neutrophil-activating protein (NAP-1) is also chemotactic for T lymphocytes. *Science* 243 1464–1466. 10.1126/science.2648569 2648569

[B15] Lin Y. Zhang M. Barnes P. F. (1998). Chemokine production by a human alveolar epithelial cell line in response to *Mycobacterium tuberculosis*. *Infect. Immun.* 66 1121–1126. 10.1128/IAI.66.3.1121-1126.1998 9488404 PMC108024

[B16] Mensah G. I. Addo K. K. Tetteh J. A. Sowah S. Loescher T. Geldmacher C. (2014). Cytokine response to selected MTB antigens in Ghanaian TB patients, before and at 2 weeks of anti-TB therapy is characterized by high expression of IFN-γ and Granzyme B and inter- individual variation. *BMC Infect. Dis.* 14:495. 10.1186/1471-2334-14-495 25209422 PMC4180837

[B17] Naumann M. Wessler S. Bartsch C. Wieland B. Meyer T. F. (1997). *Neisseria gonorrhoeae* epithelial cell interaction leads to the activation of the transcription factors nuclear factor kB and activator protein 1 and the induction of inflflammatory cytokines. *J. Exp. Med.* 186 247–258. 10.1084/jem.186.2.247 9221754 PMC2198971

[B18] Nemes E. Rozot V. Geldenhuys H. Bilek N. Mabwe S. Abrahams D. (2017). Optimization and interpretation of serial QuantiFERON testing to measure acquisition of *Mycobacterium tuberculosis* infection. *Am. J. Respir. Crit. Care Med.* 196 638–648. 10.1164/rccm.201704-0817OC 28737960 PMC5620669

[B19] Petruccioli E. Petrone L. Vanini V. Sampaolesi A. Gualano G. Girardi E. (2013). IFNgamma/TNFalpha specifc-cells and efector memory phenotype associate with active tuberculosis. *J. Infect.* 66 475–486. 10.1016/j.jinf.2013.02.004 23462597

[B20] Riedel D. D. Kaufmann S. H. (1997). Chemokine secretion by human polymorphonuclear granulocytes after stimulation with *Mycobacterium tuberculosis* and lipoarabinomannan. *Infect. Immun.* 65 4620–4623. 10.1128/iai.65.11.4620-4623.1997 9353042 PMC175663

[B21] Ruhwald M. Bodmer T. Maier C. Jepsen M. Haaland M. B. Eugen-Olsen J. (2008). Evaluating the potential of IP-10 and MCP-2 as biomarkers for the diagnosis of tuberculosis. *Eur. Respir. J.* 32 1607–1615. 10.1183/09031936.00055508 18684849

[B22] Schröder J. M. Mrowietz U. Morita E. Christophers E. (1987). Purifification and partial biochemical characterization of a human monocyte-derived, neutro phil-activating peptide that lacks interleukin-1 activity. *J. Immunol.* 139 3474–3483. 2824608

[B23] Silva Miranda M. Breiman A. Allain S. Deknuydt F. Altare F. (2012). The tuberculous granuloma: an unsuccessful host defence mechanism providing a safety shelter for the bacteria? *Clin. Dev. Immunol.* 2012:139127. 10.1155/2012/139127 22811737 PMC3395138

[B24] Subauste M. C. Jacoby D. B. Richards S. M. Proud D. (1995). Infection of a human respiratory epithelial cell line with rhinovirus: induction of cytokine release and modulation of suceptibility to infection. *J. Clin. Invest.* 96 549–557. 10.1172/JCI118067 7615827 PMC185229

[B25] Tagmouti S. Slater M. Benedetti A. Kik S. V. Banaei N. Cattamanchi A. (2014). Reproducibility of interferon gamma (IFN-γ) release assays. A systematic review. *Ann. Am. Thorac. Soc.* 11 1267–1276. 10.1513/AnnalsATS.201405-188OC 25188809 PMC5469356

[B26] van Zyl-Smit R. N. Pai M. Peprah K. Meldau R. Kieck J. Juritz J. (2009). Within-subject variability and boosting of T-cell interferon-gamma responses after tuberculin skin testing. *Am. J. Respir. Care Med.* 180 49–58. 10.1164/rccm.200811-1704OC 19342414

[B27] Verrall A. J. Netea M. G. Alisjahbana B. Hill P. C. van Crevel R. (2014). Early clearance of *Mycobacterium tuberculosis*: a new frontier in prevention. *Immunology* 141 506–513. 10.1111/imm.12223 24754048 PMC3956425

[B28] Walz A. Peveri P. Aschauer H. Baggiolini M. (1987). Purifification and amino acid sequencing of NAF, a novel neutrophil-activating factor produced by monocytes. *Biochem. Biophys. Res. Commun.* 149 755–761. 10.1016/0006-291x(87)90432-3 3322281

[B29] Wickremasinghe M. I. Thomas L. H. Friedland J. S. (1999). Pulmonary epithelial cells are a source of IL-8 in the response to *Mycobacterium tuberculosis*: essential role of IL-1 from infected monocytes in a NF-kappa B-dependent network. *J. Immunol.* 163 3936–3947. 10490995

[B30] World Health Organization (2020a). *Global Tuberculosis Report 2020.* Geneva: World Health Organization.

[B31] World Health Organization (2020b). *WHO Consolidated Guidelines on Tuberculosis, Module 1: Prevention Tuberculosis Preventive Treatment.* Geneva: World Health Organization. 32186832

[B32] Xin H. Cao X. Zhang H. Liu J. Pan S. Li X. (2020). Dynamic changes of interferon gamma release assay results with latent tuberculosis infection treatment. *Clin. Microbiol. Infect.* 26 1555.e1–1555.e7. 10.1016/j.cmi.2020.02.009 32062048

[B33] Xin H. Zhang H. Cao X. Li X. Li M. Feng B. (2019). Serum level of IL-8 is associated with reversion of QuantiFERON-TB gold in-tube tests. *J. Infect.* 78 292–298. 10.1016/j.jinf.2018.08.010 30138640

[B34] Yoshimura T. Matsushima K. Oppenheim J. J. Leonard E. J. (1987). Neutrophil chemotactic factor produced by lipopolysaccharide (LPS)- stimulated human blood mononuclear leukocytes: partial characterization and separation from interleukin 1 (IL-1). *J. Immunol.* 139 788–793. 3298433

[B35] Zhang H. Cao X. Xin H. Liu J. Pan S. Guan L. (2020). Serum level of IL-1ra was associated with the treatment of latent tuberculosis infection in a Chinese population. *BMC Infect. Dis.* 20:330. 10.1186/s12879-020-05047-x 32384874 PMC7206663

[B36] Zhang H. Xin H. Li X. Li H. Li M. Feng B. (2018). Reversion of QuantiFERON-TB Gold In-Tube test in individuals with and without prophylactic treatment for latent tuberculosis infection: a systematic review and meta-analysis. *J. Infect.* 77 276–‘282. 10.1016/j.jinf.2018.04.009 29746953

[B37] Zhang Y. Broser M. Cohen H. Bodkin M. Law K. Reibman J. (1995). Enhanced interleukin-8 release and gene expression in macrophages after exposure to *Mycobacterium tuberculosis* and its components. *J. Clin. Invest.* 95 586–592. 10.1172/JCI117702 7860742 PMC295520

